# Case of Poisoning by Camphor

**Published:** 1847-06

**Authors:** E. O. Brown

**Affiliations:** Brandenburg, Kentucky


3. *Case of Poisoning by Camphor.*—By Dr. E. O. Brown, of Brandenburg, Kentucky.

Mr. A., a stout robust man, on the 27th January, 1847, bought an ounce of gum camphor, had it put up in paper as usual, placed it in his pocket, and went to church. While there he would frequently pinch off small pieces and chew and swallow them, not noticing the quantity taken. After church he, with his father and brother, left town for home. When they had proceeded about one mile on their way, the two brothers were riding together, when suddenly the one who had taken the camphor drew up his bridle as though he was going to stop his horse, threw himself back and fell to the ground. Upon going to his assistance they found that he was powerfully convulsed; in a short time a second and a third convulsion followed. A gentleman passing at the time who was in the habit of bleeding, bled him, conveyed him to the nearest house, placed him in a warm bath, and gave him some medicine. He remained speechless, and perfectly unconscious of all that was going on for several hours. After some hours he gradually recovered his speech, but stated that he could not recollect any of the transactions of the evening on which the accident happened. He remained stupid, languid, and rather wandering all next day, but gradually recovered his former condition, and has enjoyed his health and spirits as usual since.

The foregoing history I derived from the father of the individual affected. The weight of the camphor sold by the druggist was ascertained, and on weighing it again it appeared that it had lost one hundred and ten grains. It may be concluded, therefore, that the young man swallowed something like that amount of the substance.—*West. Jour. of Medicine and Surgery.*

